# *Niviventer confucianus sacer* (Rodentia, Muridae) is a distinct species based on molecular, karyotyping, and morphological evidence

**DOI:** 10.3897/zookeys.959.53426

**Published:** 2020-08-14

**Authors:** Yaoyao Li, Yiqiao Li, Haotian Li, Jing Wang, Xiaoxiao Rong, Yuchun Li

**Affiliations:** 1 Marine College, Shandong University (Weihai), Weihai, Shandong 264209, China Shandong University Weihai China

**Keywords:** Distinct species, karyotype, molecular phylogeny, morphology, species delimitation

## Abstract

*Niviventer confucianus sacer* Thomas, 1908, which has been regarded as a subspecies of *N. confucianus*, was found to be a distinct species from *N. confucianus* based on molecular, karyotyping, and morphological characteristics in this study. *Niviventer c. sacer* was found to belong to a distinct phylogenetic clade in phylogenetic tree constructed using the mitochondrial gene *Cytb*, it clustered with *N. bukit* (Bonhote, 1903) from Vietnam and *N. confucianus* (Milne-Edwards, 1871) from Yunnan, but showed a distant relationship with *N. confucianus* from adjacent areas. The genetic distance between *N. c. sacer* and *N. confucianus* was more than 5.8%, reaching the level of interspecific differentiation. The species delimitation indicates that *N. c. sacer* is a monophyletic group. The karyotype of *N. c. sacer* (FN = 55, 8m+4st+32t+X(sm)Y(t)) differed from that of *N. confucianus* (FN = 59, 6m+4sm+2st+32t+X(sm)Y(t)). In terms of morphological features, the length of incisive foramen (LIF) and length of auditory bulla (LAB) of *N. c. sacer* is significantly larger than that of *N. confucianus* and *N. bukit* (*P* < 0.05) and the proportion of white tail tip to total tail length is significantly longer at *N. c. sacer* (≥ 1/3) than that at *N. confucianus* (≤ 1/3). Therefore, integrated analysis confirmed that *N. c. sacer* is a distinct species of genus *Niviventer* rather than a subspecies of *N. confucianus* or *N. bukit*, namely *N. sacer*, which is only distributed in Shandong.

## Introduction

*Niviventer confucianus sacer*Thomas (1908) was named based on specimens collected from Mount Ai, Yantai, Shandong, China (type locality) according to its different morphology from *N. confucianus* in other areas. The holotype (NHMUK 8.2.8.8) is preserved in the Natural History Museum, London (NHMUK). Thomas (1908) described it as a buff-grey subspecies of *N. confucianus*, with the tail long-haired and white-tipped. However, the taxonomic status of *N. c. sacer* remains controversial. These species were originally classified in the genus *Mus* and later in *Rattus* (Thomas 1908; Allen 1926), but the genus name was changed to *Niviventer*, established by Musser (1981). *Niviventer c. sacer* has been adopted as a subspecies of *N. confucianus* by taxonomists based on morphology. Allen (1940) considered *N. c. sacer* as one of the four subspecies of *N. confucianus*; Wang and Zheng (1981) divided *N. confucianus* into six subspecies and pointed out that *N. c. sacer* were distributed in Shandong, Shanxi, Shaanxi, and Gansu of central China; Huang et al. (1995) identified *N. c. sacer* as one of the eight subspecies of *N. confucianus*; Smith and Xie (2009) extended the view of Wang (2003) and considered that *N. confucianus* distributed in most northern areas of the Yangtze River in China were *N. c. sacer*.

In terms of molecular phylogeny, Zhang et al. (2016) estimated phylogenetic relationships using topotype specimens of white-bellied rats in China based on multi-locus analysis and firstly used five specimens from Yantai, Shandong. They found that *N. c. sacer* formed an independent clade in the phylogenetic tree and a sister clade with *N. confucianus* and *N. bukit*, indicating that *N. c. sacer* is a sister species or branch of *N. confucianus*. However, Zhang et al. (2016) considered that this genetic difference did not reach the species level. Ge et al. (2018a, b) analyzed a larger number of specimens to evaluate the internal differentiation of *N. confucianus* based on the above study, the results showed that *N. c. sacer* from Shandong were not clustered within the clades of *N. confucianus*, but were most closely related to *N. confucianus* from Yunnan and *N. bukit* from Vietnam. Therefore, they considered that *N. c. sacer* might not be a member of *N. confucianus*, but rather *N. bukit*. Karyotyping studies have shown that the chromosomal fundamental arm number (FN) and karyotypes of *N. confucianus* from Shandong were significantly different from those from Guangdong, Shaanxi, and Thailand. The karyotype of *N. confucianus* from Shandong (*N. c. sacer*) was 2n = 46, FN = 55, 8m+4st+32t+X(sm)Y(t), whereas that from Thailand was 2n = 46, FN = 58, 6m+6sm+2st+30t+X(t)+Y(t); that from Guangdong was 2n = 46, FN = 54, 6m+38t+X(sm)Y(t); and that from Shaanxi was 2n = 46, FN = 58, 8m+2sm+2st+32t+X(sm)Y(t) (Jiang 1995; Wang et al. 1997; Wang et al. 2003).

The Shandong Peninsula is surrounded on three sides by the Bohai and Huanghai seas. The southwest mountain area and east hill area are isolated by consecutive plains of North China Plain and middle and lower Yangtze River plain. Also, the plain separates mountain habitats in Shandong from those in adjacent areas, forming unique topographical features. Between the southwest mountains and east hills in Shandong is the Jiaolai Plain, which forms an inner isolated area. Studies showed that the unique topographical features in Shandong resulted in the development of endemic species. For example, *Rana kunyuensis* is only distributed in Mount Kunyu in Yantai, Shandong, and *Pseudohaplotropis culaishanica* is only distributed in Mount Culai in Shandong. These new species are unique to the habitats of Shandong and were discovered in recent years (Lu and Li 2002; Cao and Yin 2008). More detailed study is needed to understand the diversity of the region.

Molecular methods are effective for identifying sister species with similar appearance and detecting cryptic species in species complexes (Francis et al. 2010; Chen et al. 2011; Lu et al. 2015; Zhang et al. 2016; Li et al. 2019). Geometric morphometrics is a statistics-based quantitative way of comparing shape (morphology) across different specimens. It not only offers the ability to describe shape precisely and accurately, but also facilitates visualization and interpretation of results of the analysis. With the development and improvement of geometric morphometry, it has become an important method to study the morphological differentiation between species and within species, and has been widely used in rodents (Cardini and O’Higgins 2004; Renaud and Michaux 2007; Lu et al. 2015; Alhajeri 2018). In this study, we systematically reassessed the taxonomic status of *N. c. sacer* by using the mitochondrial *Cytb* sequence as a molecular marker to analyze phylogenetic relationships. We also used the automatic barcode gap discovery (ABGD) method to define the species status combined with karyotyping results, morphological characteristics, and measurement indices.

## Materials and methods

### Sample collection and ethics

**Figure 1.**
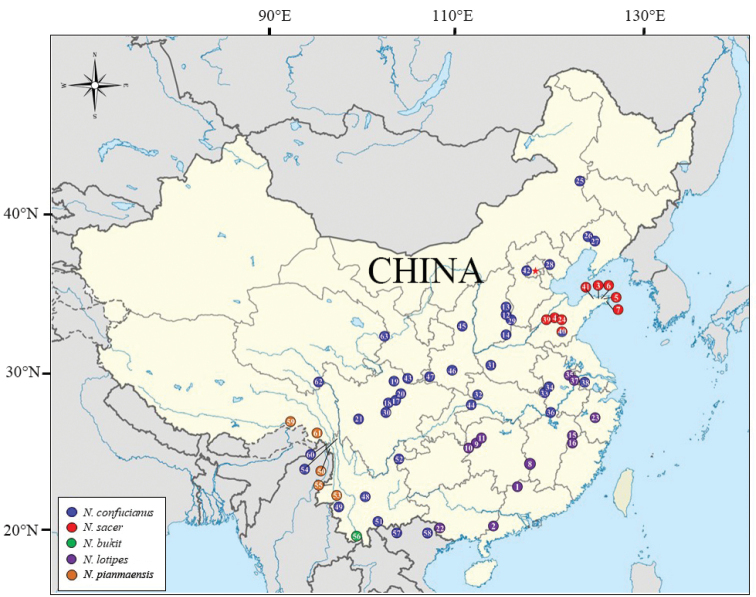
Distribution of phylogenetic clades of *N. confucianus* species complex obtained from *Cytb*. The numbers correspond to the locality code in Suppl. material 1, Table S1.

A total of 214 specimens of *N. confucianus* species complex was collected from 35 sampling sites in China using Sherman living cages from March 2009 to August 2018. The sampling sites covered the distribution range of *N. confucianus* (Fig. 1), and sample information is shown in Suppl. material 1: Table S1. Chromosome preparations were made by the live bone marrow method (Searle 1986). All specimens including the pelt, carcass, and skull were stored at Shandong University (Weihai). All animal sample collection protocols complied with the current laws of China and all animals were handled in a manner consistent with the guidelines approved by the American Society of Mammalogists (Sikes et al. 2016).

### DNA sequencing

DNA was extracted from muscle samples using the Easy Pure Genomic DNA Kit (TransGen Biotech Co., Ltd., Beijing, China). The complete mitochondrial cytochrome *b* gene (*Cytb*, 1140 bp) was amplified by PCR using the primers described by Irwin et al. (1991). The primer sequences were as follows: Nivicob1 (5'-TGTCATTATTTCTACACAGCACTTA-3') and Nivicob2 (5'-TTTGGGTGTTGATGGTGGG-3'). PCR was performed in a volume of 50 μL containing 30 ng template DNA, 2×EasyTaq PCR SuperMix 25 μL, and 0.5 μM primers. The thermocycling protocol was as follows: initial denaturation of 4 min at 94 °C; 32 cycles of 94 °C for 30 s, annealing temperature (Tm) for 30 s, 72 °C for 70 s; and final extension for 6 min at 72 °C. Detection was carried out by 1% agarose gel electrophoresis, and PCR products were directly sequenced by Sanger sequencing.

### Phylogenetic analysis

We supplemented our new *Cytb* data with homologous sequences (>1,140 bp) of *Niviventer* available in GenBank (Balakirev and Rozhnov 2010; Chen et al. 2011; Balakirev et al. 2010; Zhang et al. 2016). Here, six species were selected as outgroup taxa: *Bandicota indica*, *Berylmys bowersi*, *Leopoldamys nielli*, *Maxomys surifer*, *Rattus norvegicus*, and *Rattus andamanensis* (Michaux et al. 2007; Laredo et al. 2011; Balakirev et al. 2012, 2013; Koma et al. 2013; Conroy et al. 2013). GenBank accession numbers for the original sequences used in this study are MT333860-MT334073 (Suppl. material 1: Table S1).

All sequences were aligned with Clustal X 2.0 (Larkin et al. 2007), manually edited in BioEdit 7.2.5 (Hall 1999), and corrected to eliminate interference from degenerate bases. We used the Akaike Information Criterion (AIC) in jModeltest 1.0 (Posada 2008) to select the best-fit model of sequence evolution for locus alignment. We calculated the population haplotypes in DnaSP (Librado and Rozas 2009) and performed phylogenetic reconstructions based on *Cytb* using the maximum likelihood (ML) and neighbor-joining (NJ) approaches with the TN93+G+I model and K2P model in MEGA 7 (Kumar et al. 2016), respectively. Bootstraps were obtained using a rapid bootstrapping algorithm with 1000 replicates. We also constructed a phylogenetic tree using Bayesian inference (BI) in MrBayes 3.2 (Ronquist et al. 2012, Balakirev et al. 2013) based on the TN93 model. This step was repeated twice, the replacement rate of the sequence with invgamma was determined, and the process was conducted four Markov chain Monte Carlo runs with four chains for 10 million generations, sampling every 1000 trees and discarding the first 25% as burn-in. We calculated Kimura-2-parameter (K2P) distances of *Cytb* in MEGA 7 (Kumar et al. 2016) for pairwise comparisons of genetic differentiation within and between different phylogenetic lineages, and standard error was analyzed using 1000 bootstrap tests.

### Species delimitation

We used ABGD (Puillandre et al. 2012) to recover candidate species. All aligned haplotype sequences (166 *Cytb*) were uploaded to the web interface (http://wwwabi.snv.jussieu.fr/public/abgd/abgdweb.html) and run with the following settings: P (prior limit to intraspecific diversity) range of 0.001–0.1 and relative gap widths (X) of 0.5, 1.0, 1.5, 2.0, and 2.5. Transition/transversion bias (TS/TV) was estimated using MEGA 7. We selected the Kimura 80 model to analyze our data and set the number of both steps and bins to 25.

### Karyotype analysis

We captured and analyzed the mitotic phase with improved chromosome dispersion using Cytovision System (Applied Imaging, Newcastle upon Tyne, UK). The diploid number (2n) and chromosome fundamental arm number (FN) were determined in each karyotype. Chromosomes were classified according to Levan et al. (1964) and Motokawa et al. (2001) to analyze the differences in karyotypes among different clades.

### Morphological analysis

To understand the morphological diversity of *N. confucianus*, *N. c. sacer*, *N. bukit* and *N. lotipes*, which are closely related species in *N. confucianus* species complex, we explored differences in external and skull morphology among the four species, in which the measurements data of *N. bukit* was reference to Ge et al. (2018b), the other three were collected and measured by our laboratory. We determined four external indices (head and body length (HBL); tail length (TL); ear length (EL); hind foot length (HFL)) and eight skull indices (greatest length of skull (LS); zygomatic width (ZW); interorbital breadth (IOB); breadth of rostrum (BR); length of incisive foramen (LIF); length of upper tooth row (LUTR); length of auditory bulla (LAB); length of upper diastema (LD)) according to Huang et al. (1995), Yang et al. (2005), Xia et al. (2006), and Ge et al. (2018b). Skull indices were measured with a digital vernier caliper (0.01 mm). Adult specimens were identified by the full eruption of the molars and the frequency histogram of head and body length (Yang 1990, Li et al. 1989, 1990). Male and female specimens were mixed in analyses since previous studies identified no significant differences in the external and cranial measurements between sexes (Stefen and Rudolf 2007; Yang et al. 2011).

To characterize the differences among the four species, standard statistics including the mean and standard error were applied. Pairwise differences between major species groups were tested by an analysis of variance (ANOVA) using least significant difference (LSD) tests, as LSD is more commonly used and sensitive to obtain statistical differences, and multivariate analysis (principal component analysis, discriminant analysis, cluster analysis) was also performed. These analyses were performed using SPSS Statistics 24.0 (SPSS, Chicago, IL, USA).

We photographed the skulls of specimens of *N. confucianus*, *N. c. sacer* and *N. lotipes* for quantitative analysis (as *N. bukit* was not sampled). The dorsal, ventral, and lateral sides of the skull and lateral view of the mandible were analyzed separately. Landmarks are homologous site of geometric morphology with biological significance on the specimen, which were selected to reflect the shape of the mandible (Bookstein 1991). Landmarks and semi-landmarks were digitized in tpsDig 2.30 (Rohlf 2017), and their location is shown in Suppl. material 2: Figure S1. The raw datasets for each of the above four views were examined to evaluate whether the specimens greatly deviated from the average values. Generalized procrustes analysis (GPA) was used to carry out the superimposition of landmarks, in order to remove shape-irrelevant variables like size, orientation and position from the original landmark configurations, leaving the real shape information. This is a necessary step in geometric morphometric analysis (Gower 1975, Rohlf and Slice 1990). Relative distortion analysis was performed with tpsRelw, and the relative warp score (RW) was determined. Principal component analysis was conducted to visualize shape differences between individuals, and thin-plate spline transformation grids of extreme value were used to show skull shape differences (Bookstein, 1997; Slice, 2007).

We also analyzed the dorsal hair color, spiny-ness of hairs, yellow patches, and white tail tip of *N. c. sacer*, *N. confucianus* and *N. lotipes* to compare external morphological features by Chi-square test. The dorsal hairs color and spiny-ness of hairs were identified by observing and touching on pelage of specimens on three-category records, as the color of dorsal hairs were all brown, tan and all yellow; the spiny-ness of hairs were hard, medium and soft; the yellow patches on the chest observed by direct observations of pelage specimen with confirmation of specimen photos, which were recorded by dichotomy, yes or no; and the white tail tip was calculated based on the ratio of the measured tail tip length to the total tail length, divided into 4 ranks: 0, 1/4, 1/3, 1/2.

## Results

### Sequence data

We obtained 1140 bp of mitochondrial *Cytb* sequences from 312 individuals with 166 haplotypes in this study. Among them, there were 708 conserved loci, 395 parsimoniously informative loci, and 37 single-variant loci; no insertion, deletion, or termination codons were found. The transition/transversion bias was 5.40, and nucleotides in all sequences were accurately translated into amino acids.

### Phylogenetic relationships

**Figure 2.**
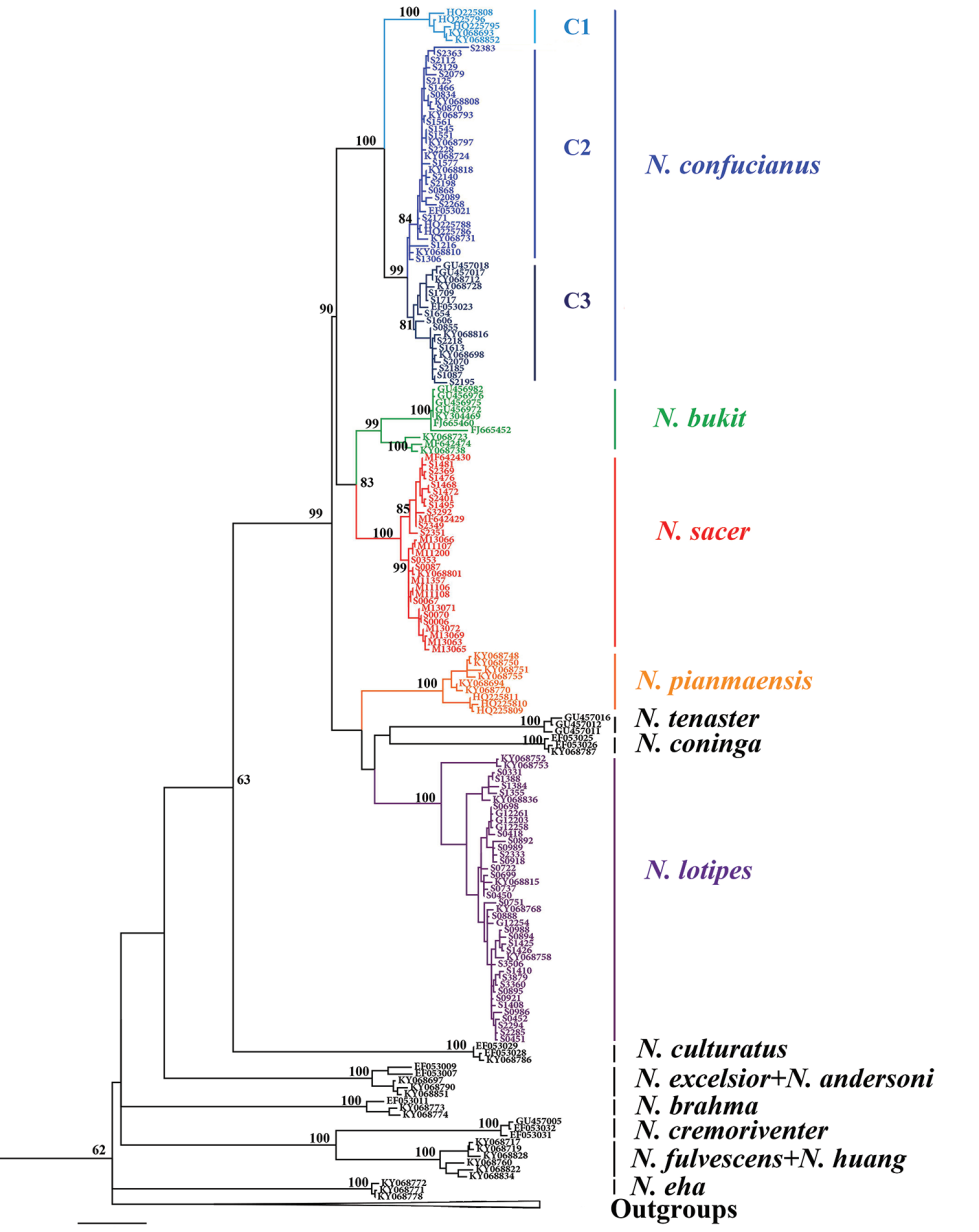
Phylogenetic analyses of *Cytb* gene from all haplotypes by maximum likelihood.

The phylogenetic trees constructed based on haplotype data using the NJ, ML, and BI methods showed essentially the same topology with high confidence values (Fig. 2, Suppl. material 2: Figs S2, S3): (1) the *N. confucianus* individuals clustered into three well-supported clades (i.e., clades C1, C2, C3). Clade C1 is distributed in southwestern Yunnan and southeastern Tibet; Clade C2 is found from central to northern China, and includes two haplotypes of Linyi and Zibo from Shandong; Clade C3 extended from the north of Southeast Asia to central China. (2) *N. c. sacer* split into two deep subclades in the central Shandong and Yantai, Weihai regions; interestingly, *N. bukit* from Vietnam and two haplotypes from Xishuangbanna in Yunnan clustered into one clade, forming a sister clade to *N. c. sacer*.

According to the constructed phylogenetic relationship, the K2P genetic distances within and between each clade were calculated. Genetic distances were found to range from 0.011 to 0.022 within the four clades and 0.053 to 0.084 between the four clades (*N. confucianus*, *N. sacer*, *N. lotipes*, and *N. bukit*). Among them, the genetic distance between *N. c. sacer* and *N. bukit* showed the lowest value (0.053); however, the genetic distance between *N. c. sacer* and other species was higher than 0.058 (Table 1).

**Table 1.** Genetic distance of *Niviventer* calculated based on cytochrome *b* (*Cytb*). Estimates of evolutionary divergence (with SEs) over clades are given in the lower triangle, within-clades distances are given in the diagonal. Abbreviations for different species: CONF, *N. confucianus*; SAC, *N. sacer*; LOT, *N. lotipes*; PIA, *N. pianmaensis*; TEN, *N. tenaster*; ANEX, *N. andersoni+N. excelsior*; BRA, *N. brahma*; BUK, *N. bukit*; CON, *N. coninga*; CRE, *N. cremoriventer*; CUL, *N. culturatus*; EHA, *N. eha*; FUHU, *N. fulvescens+N. huang.*

	CONF	SAC	LOT	PIA	TEN	ANEX	BRA	BUK	CON	CRE	CUL	EHA	FUHU
CONF	0.018±0.002												
**SAC**	**0.058**±**0.006**	0.011±0.002											
LOT	0.077±0.007	0.084±0.008	0.015±0.002										
GLA	0.073±0.007	0.076±0.007	0.079±0.007	0.016±0.002									
TEN	0.094±0.009	0.092±0.009	0.087±0.009	0.089±0.009	0.008±0.002								
ANEX	0.134±0.010	0.133±0.010	0.129±0.010	0.136±0.011	0.142±0.011	0.019±0.003							
BRA	0.129±0.010	0.135±0.011	0.132±0.008	0.140±0.011	0.149±0.012	0.154±0.012	0.016±0.003						
**BUK**	0.064±0.006	**0.053**±**0.006**	0.082±0.008	0.071±0.007	0.096±0.009	0.135±0.010	0.136±0.010	0.022±0.003					
CON	0.095±0.009	0.100±0.009	0.088±0.012	0.090±0.009	0.102±0.010	0.140±0.011	0.151±0.011	0.092±0.009	0.004±0.001				
CRE	0.154±0.012	0.149±0.011	0.161±0.011	0.159±0.012	0.172±0.013	0.152±0.011	0.149±0.012	0.154±0.012	0.160±0.012	0.007±0.003			
CUL	0.119±0.010	0.129±0.011	0.133±0.011	0.128±0.011	0.153±0.013	0.137±0.011	0.151±0.012	0.128±0.011	0.140±0.012	0.147±0.011	0.004±0.001		
EHA	0.143±0.011	0.134±0.011	0.146±0.011	0.141±0.011	0.157±0.011	0.141±0.011	0.149±0.012	0.145±0.011	0.133±0.011	0.160±0.012	0.140±0.012	0.004±0.001	
FUHU	0.158±0.012	0.153±0.012	0.142±0.011	0.150±0.011	0.159±0.012	0.157±0.012	0.142±0.012	0.158±0.012	0.152±0.011	0.095±0.009	0.152±0.013	0.140±0.011	0.019±0.003

### Species delimitation

Five ABGD analyses with different relative gap width values (X = 0.5, 1, 1.5, 2, and 2.5) were performed on 166 *Cytb* sequences, and two gaps (distance = 0.05 and 0.11) were observed (Suppl. material 2: Fig. S4). All analyses consistently supported a 17-group scenario when intraspecific divergence(p) = 0.001-0.0083 (Suppl. material 2: Table S2).

**Figure 3.**
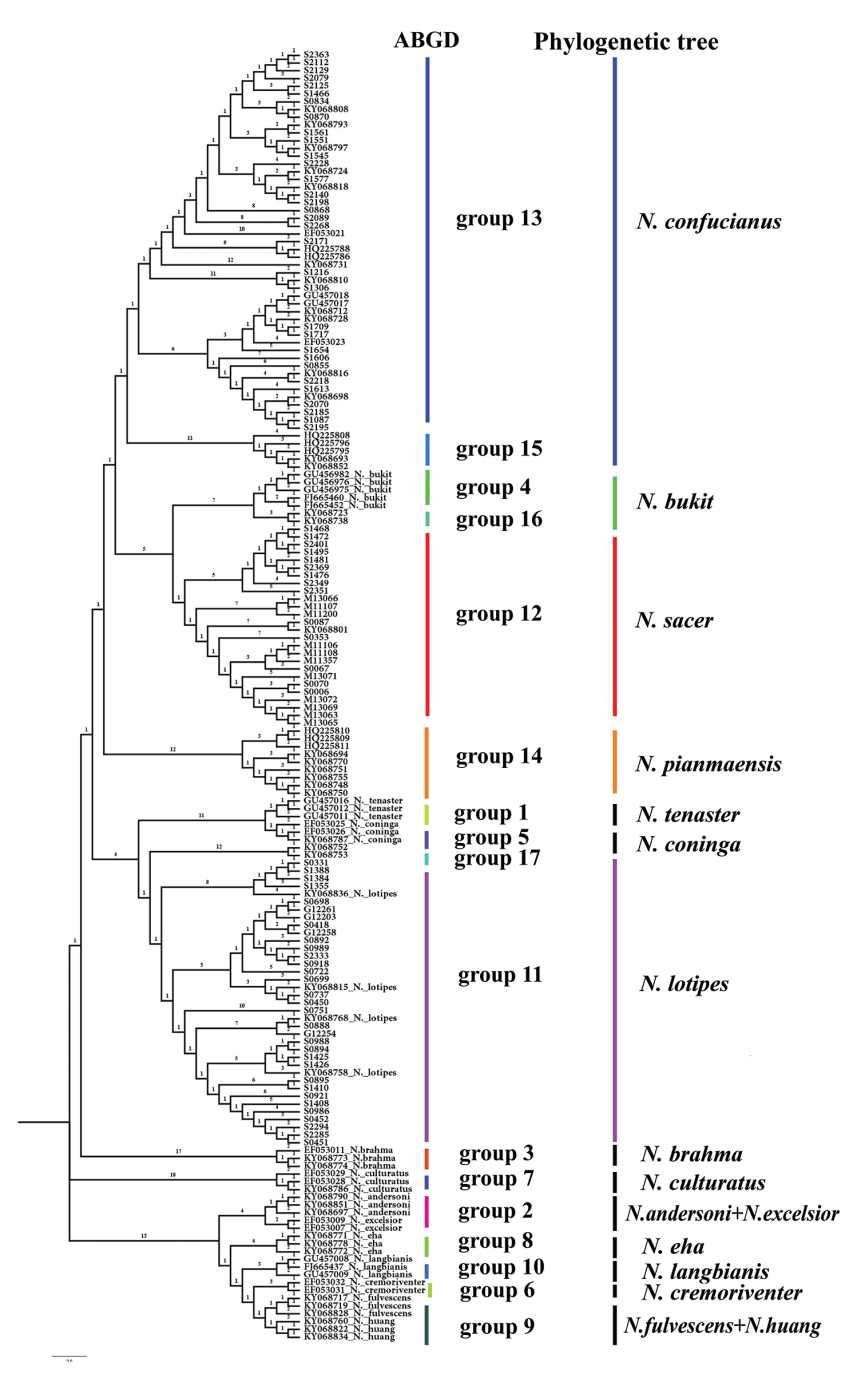
Comparison of ABGD species tree based on genetic distance and phylogenetic tree.

The species tree constructed by ABGD based on genetic distance is shown in Figure 3. The *N. confucianus* specimens are included in seven groups (groups 11–17), the haplotypes of *N. c. sacer* appeared as a monophyletic group (group 12), and the haplotypes of *N. confucianus* from Xishuangbanna in the Yunnan (group 16) and *N. bukit* from Vietnam (group 4) corresponded to *N. bukit* clade in the phylogenetic tree.

### Karyotype analysis

**Figure 4.**
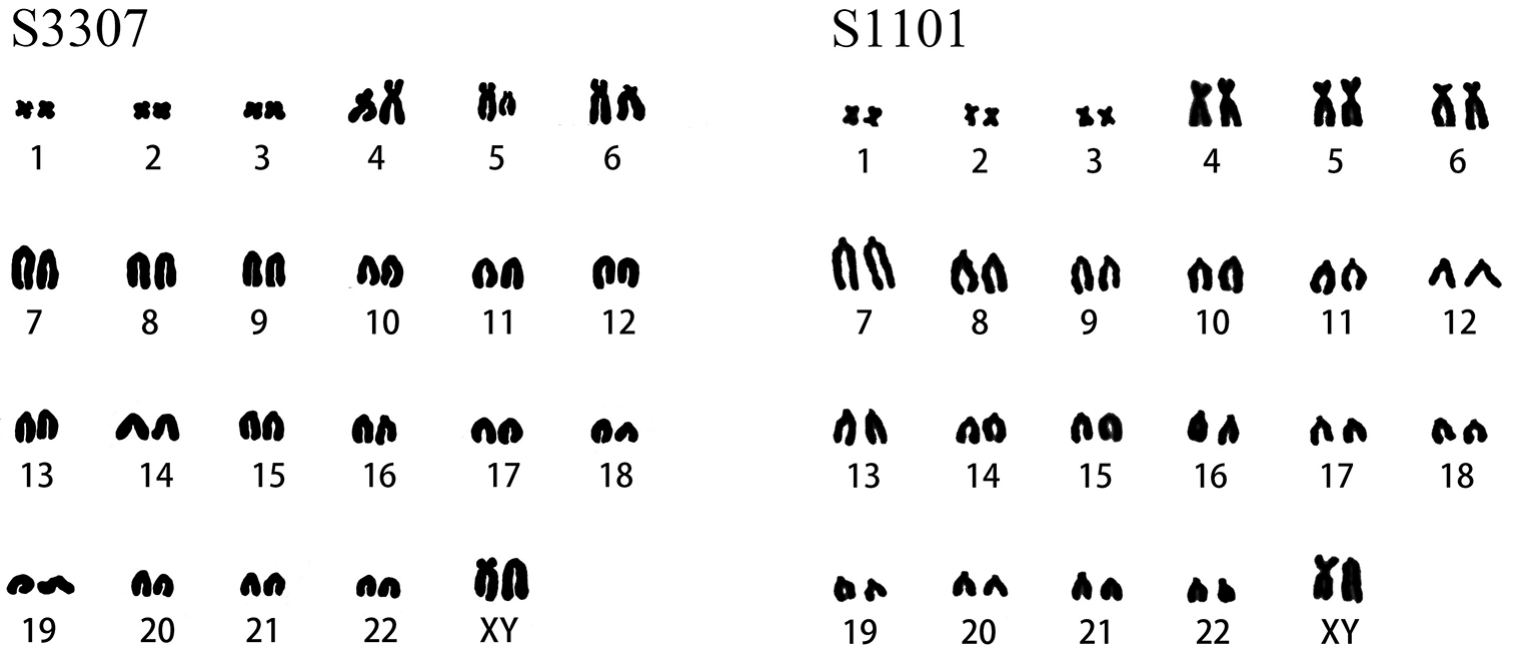
Karyotype of *N. sacer* (S3307) and *N. confucianus* (S1101).

Karyotype analysis showed that the karyotype of *N. c. sacer* (♀2, ♂4) differed from that of *N. confucianus* (♂3). The diploid number (2n) is 46 for both, but the karyotype characteristics of *N. c. sacer* is FN = 55, 8m+4st+32t+X(sm)Y(t), chromosome composition: four pairs with metacentric chromosomes, two pairs with subtelocentric chromosomes, 16 pairs telocentric chromosomes, and two sex chromosomes; in contrast, the karyotype of *N. confucianus* is FN = 59, 6m+4sm+2st+32t+X(sm)Y(t), chromosome composition: three pairs with metacentric chromosomes, two pairs with submetacentric chromosomes, one pair with subtelocentric chromosomes, 16 pairs telocentric chromosomes, and two sex chromosomes (Fig. 4).

### Morphological analysis

A total of 98 adult individuals of *N. confucianus*, *N. c. sacer* and *N. lotipes* was screened by age identification, and the complete external indices of 84 individuals and skull indices of 72 individuals were obtained. Most characteristics showed normal distributions (*P* > 0.05), and thus we performed parametric statistics analysis (Suppl. material 2: Table S3). Information on general variation in body form of the four species is given in Table 2 and Suppl. material 2: Tables S4 and S5. The ANOVA results showed significant morphological differences between the species in external indices and most skull indices (Suppl. material 2: Table S4). Multiple comparison results showed the skull indices (LIF and LAB) of *N. c. sacer* are significantly larger than those of other three species (Table 2, Suppl. material 2: Table S5).

**Table 2.** External and craniodental measurements (mean±1 *SD*, range) of *N. sacer*, *N. bukit*, *N. confucianus*, and *N. lotipes* in China. HBL = head and body length; TL = tail length; EL = ear length; HFL = hind foot length; LS = greatest length of skull; ZW = zygomatic width; IOB = interorbital breadth; BR = breath of rostrum; LIF = length of incisive foramen; LUTR = length of upper tooth row; LAB = length of auditory bulla; LD = length of upper diastema.

Indices	*N. sacer*			*N. bukit*			*N. confucianus*			*N. lotipes*		
	*n*	Mean ± *SD*	Min ~ Max	*n*	Mean ± *SD*	Min ~ Max	*n*	Mean ± *SD*	Min ~ Max	*n*	Mean ± *SD*	Min ~ Max
HBL	47	146.55±11.38	122.00~169.00	23	135.96±10.44	120.00~157.00	25	142.18±20.07	113.00~206.00	26	142.85±15.88	108.00~172.00
TL	38	158.99±12.81	121.00~179.00	21	164.90±9.51	144.00~185.00	25	158.24±20.38	115.00~189.00	21	182.00±15.06	147.00~212.00
EL	48	20.35±1.21	17.10~22.73	22	21.89±1.45	19.00~25.00	27	20.35±1.75	16.86~23.89	26	20.39±1.35	18.21~22.85
HFL	48	28.01±1.13	25.55~30.18	23	28.11±2.11	24.50~35.00	27	26.57±1.47	24.31~29.69	26	26.93±1.47	24.02~30.66
LS	20	36.87±1.88	32.30~39.89	5	36.13±1.10	34.32~37.29	27	35.89±1.83	33.31~39.51	25	36.32±1.53	33.36~39.26
ZW	46	16.84±0.98	13.32~18.83	5	15.57±0.59	14.81~16.39	27	16.37±0.85	14.95~18.03	25	16.42±0.56	15.58~17.73
IOB	46	5.66±0.19	5.27~6.22	5	5.75±0.24	5.49~6.06	27	5.32±0.27	4.84~5.84	25	5.43±0.20	4.81~5.84
BR	46	6.18±0.36	5.43~6.79	5	5.99±0.54	5.15~6.45	27	6.11±0.45	5.08~7.09	25	6.35±0.43	5.45~7.05
LIF	46	6.98±0.43	6.10~7.98	5	5.55±0.59	4.63~6.05	27	6.16±0.52	5.22~7.31	25	6.22±0.45	5.12~6.86
LUTR	20	6.11±0.22	5.69~6.45	5	5.82±0.29	5.53~6.22	27	5.96±0.27	5.56~6.70	25	5.70±0.22	5.34~6.16
LAB	46	5.38±0.46	4.73~6.50	4	4.90±0.57	4.31~5.65	27	5.22±0.23	4.78~5.63	25	5.24±0.26	4.88~5.74
LD	46	9.60±0.56	8.60~10.76	5	9.44±0.76	8.46~10.42	27	9.02±0.71	8.07~10.89	25	9.37±0.50	8.46~10.43

**Figure 5.**
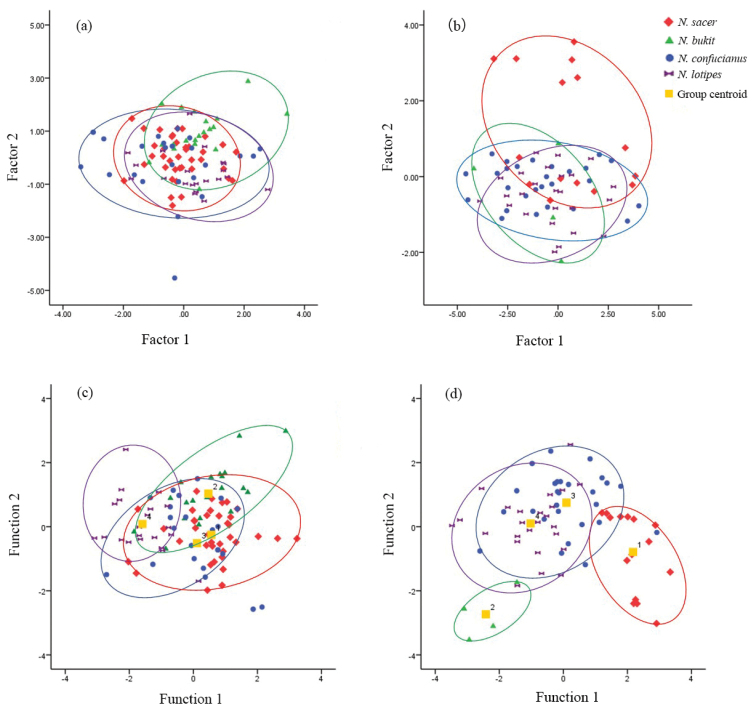
Principal component analysis and discriminant analysis of external and skull morphological indices. Principal component plots of external and skull indices are shown in **a** and **b**. Discriminant function plots of external and skull indices are shown in **c** and **d**, respectively.

In the PCA of external indices, two factors had eigenvalues exceeding 1.0, and the first two axes captured 36.6% and 27.6% of the total variation, accordingly (Table 3). The TL and EL provided the greatest contribution to the factor loadings of PC1(0.764 and 0.619, respectively; Table 3). PC2 was mainly influenced by HBL (-0.711; Table 3). The main scatter plots showed that the specimens from the four species had greatly mixed external indices, indicating they cannot be distinguished by the external measurement method alone (Fig. 5a). In the PCA of skull indices, the first two axes captured 54.1% and 16.4% of the total variation, accordingly. LS, LD and LIF are the three measurements that have the highest correlation with PC1(0.936, 0.875 and 0.874, respectively; Table 3), which is obviously associated with size, as all the measurements have the same sign, and most have the same magnitude. PC2 was mainly influenced by LAB and BR (0.873 and -0.449, respectively; Table 3), which seems to be a shape factor given the different magnitudes and signs. The changes of length of auditory bulla and breath of rostrum are associated with PC2. The main scatter plots showed that the four species mix with each other, but part of *N. c. sacer* can separated from those of others (Fig. 5b, Table 3). In discriminant analysis, 68.3–80.3% of the individuals were correctly classified (Suppl. material 2: Table S6). The scatter plots of the discriminant function showed that individuals between clades were more likely to be confused based on external indices; however, based on skull indices, most individuals of *N. c. sacer* were accurately identified and classified, whereas a few individuals were easily confused with *N. confucianus* but completely separated from *N. bukit* and *N. lotipes* (Fig. 5c, d).

Cluster analysis of external and skull measurement indices showed that the distributions of *N. c. sacer* and other three species are mixed and mosaic in the dendrogram (Suppl. material 2: Fig. S5), indicating that *N. c. sacer* and its close relatives cannot be distinguished based on traditional morphological characteristics.

**Table 3.** Factor loadings, eigenvalues, and the variance explained by each principal component based on the external and skull measurements of *N. sacer*, *N. bukit*, *N. confucianus*, and *N. lotipes*.

	PC1	PC2
HBL	0.504	-0.711
TL	0.764	-0.277
EL	0.619	0.538
HFL	0.493	0.481
Eigenvalues	1.465	1.103
% of variance explained	36.614	27.577
LS	0.936	-0.036
ZW	0.825	-0.392
IOB	0.589	0.228
BR	0.716	-0.449
LIF	0.874	0.267
LUTR	0.589	0.271
LAB	0.184	0.873
LD	0.875	-0.012
Eigenvalues	4.329	1.315
% of variance explained	54.106	16.438

### Geometric morphometric analysis

**Figure 6.**
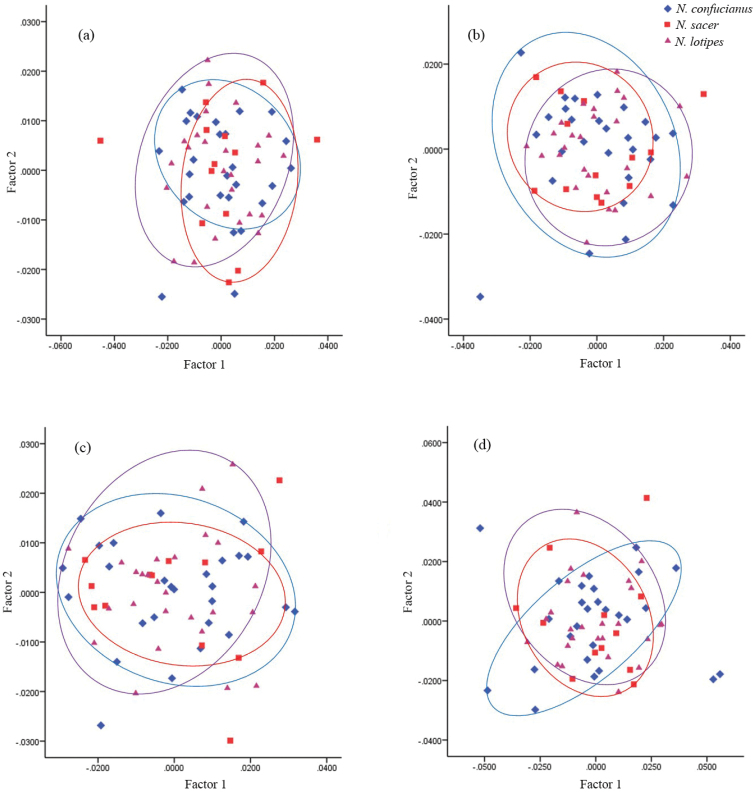
Principal component analysis of dorsal view (**a**), ventral view (**b**), lateral view (**c**) of skull, and lateral view of the mandible (**d**) of the three clades.

The average configuration of the superimposition on the dorsal, ventral, lateral view of the skull and lateral view of the mandible of the three species is shown in Suppl. material 2: Figure S6. The main scatter plots constructed using RW1 and RW2 revealed no clear line between the three clades, indicating that variation in the samples from the three clades is not obvious, and geometric morphometric cannot distinguish the samples in the three clades (Fig. 6).

**Figure 7.**
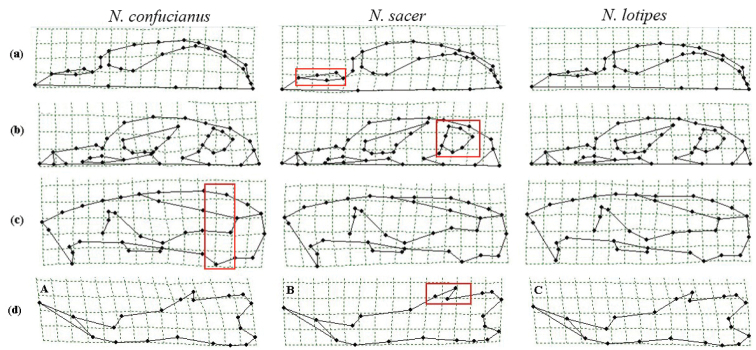
Thin plate splines of dorsal view (**a**), ventral view (**b**), lateral view (**c**) of skull, and lateral view of the mandible (**d**) of *N. sacer*, *N. confucianus*, and *N. lotipes*.

The thin-plate spline transformation grids of extreme value showed that there are some variations between the three clades (highlighted in red boxes): the maxilla of *N. c. sacer* is slightly wider than those of *N. confucianus* and *N. lotipes* in the dorsal view of the skull (Fig. 7a). The auditory vesicle of *N. c. sacer* is slightly larger than those of *N. confucianus* and *N. lotipes* in the ventral view of skull (Fig. 7b). The height of basion of *N. confucianus* is slightly higher than those of *N. c. sacer* and *N. lotipes*, and the top of the skull tended to be rounder in the lateral view of the skull (Fig. 7c). Samples from *N. c. sacer* showed a narrower coronal process of the mandibular teeth in the lateral view of the mandible (Fig. 7d). However, each deformation was not obvious enough to distinguish samples between the three species.

### External morphological features

In a comparison of the external morphological features of *N. c. sacer*, *N. confucianus*, and *N. lotipes*, we found that samples in the three clades could not be distinguished based on the dorsal hair color and yellow patches, but there were significant differences (*P* < 0.05) in the spiny hairs and white tail tip: the spiny hairs of *N. c. sacer* is softer than those in the other clades; the tail color of *N. c. sacer* is the upper brownish black, the lower white, whereas the proportion of the white tail tip is more than 1/3, which is the same as the holotype specimens first found by Thomas (1908) in Yantai, Shandong. The ventral surface of the tail in *N. confucianus* is brownish black, the proportion of the white tail tip is less than 1/3, and the tail is more often without white hairs in *N. lotipes*, with a proportion of white hair of less than 1/4 (Table 4, Suppl. material 2: Fig. S7).

**Table 4.** Comparison of external morphological features of *N. sacer*, *N. confucianus*, and *N. lotipes* in China using chi-square test.

Indices	Category	*N. confucianus*	*N. sacer*	*N. lotipes*	*χ^2^*	*P*
Dorsal hair color	all brown	13	7	14	2.900	0.575
	tan	14	16	14		
	all yellow	9	7	6		
Spiny hairs	hard	7	7	18	19.573	**0.001** ***
	medium	10	3	10		
	soft	19	20	6		
Yellow patches	no	25	25	25	1.743	0.418
	yes	11	5	9		
White tail tip	0	10	2	14	27.036	**<0.001** ***
	1/4	7	2	8		
	1/3	11	6	3		
	1/2	4	12	2		

## Discussion

### Taxonomic status of *N. c. sacer*

We analyzed specimens from across China and surrounding countries and recover evidence that *Niviventer confucianus sacer* should be elevated to *Niviventer sacer*. Molecular phylogenetic analysis indicated that *N. sacer* formed a sister branch with *N. confucianus* from Yunnan and *N. bukit* from Vietnam rather than with *N. confucianus* from adjacent areas in Shandong (Shanxi, Jiangsu, Hebei, Henan, etc.), which is consistent with the results of Ge et al. (2018a, b). *Niviventer sacer* is also distinct according to the species delimitation (ABGD method), and the genetic distance (K2P) between *N. sacer* and *N. confucianus* was as high as 5.8%, whereas that with *N. bukit* was 5.3%, both exceeding the empirical threshold of dividing rodent species (5%, Avise and Walker 1999; Baker and Bradley 2006; Zhang et al. 2019), revealing that *N. sacer* is a distinct species.

In contrast to the conclusions of Ge et al. (2018a, b), who suggested that *N. c. sacer* may be a population of *N. bukit*, we consider that they are two independent species based on the following four reasons:

(1) in terms of geographical distribution, *N. sacer* and *N. bukit* are distributed in Shandong and Vietnam, respectively, separated by a distance of more than 2,000 kilometers;

(2) Zhang et al. (2016) used nuclear genes to construct a multilocus phylogeny tree, indicating that *N. c. sacer* was nested among several geographically-adjacent subspecies of *N. confucianus* to the exclusion of *N. bukit*;

(3) Ge et al. (2018b) only used *Cytb* and the number of specimens of *N. c. sacer* was limited, and for this reason *N. c. sacer* was clustered with *N. bukit* rather than *N. confucianus*, it may be the misleading effect of rapid or saturation mutation of *Cytb*;

(4) Morphological analysis shows there are significant differences between *N. sacer* and *N. bukit* in external and skull indices.

Interestingly, *N. sacer* distributed in the east hills and southwest mountains in Shandong is divided into two independent small lineages. However, *N. confucianus* is only distributed in the southwest mountains (Mount Lu and Mount Meng) in the Shandong area and forms the same clade with *N. confucianus* from adjacent areas. Therefore, *N. sacer* and *N. confucianus* show sympatry characteristics in the southwest mountains area, but their genetic distance (5.6%) is higher than 5% and they have significantly different karyotypes and morphologies. The sympatry of *N. sacer* and *N. confucianus* in the southwest mountains indicates that they are distinct species rather than subspecies without hybridization.

Morphological analysis showed that *N. sacer*, *N. bukit*, *N. confucianus*, and *N. lotipes* had significantly different morphological characteristics, which are reflected in the larger skull, but have similar skull shapes and characteristics. The morphological characteristics of the tail are important traits for distinguishing different species and are probably associated with adaptations for an arboreal lifestyle in different forest types. Moreover, tails may play important roles in the recognition of conspecifics (Siegel 1970, Ge et al. 2018b). In a comparison of the morphological characteristics of *N. sacer* and *N. confucianus*, we found a significant difference in tail color: the upper tail color of *N. sacer* is brownish black, while the lower color is white, and approximately one-third of the tip was all white, which has been observed in holotype specimens found by Thomas (1908) in Yantai, Shandong; in contrast, the tail of most *N. confucianus* are brown-black with only a white tail tip.

The karyotype of *N. sacer* in this study is 2n = 46, FN = 55, 8m+4st+32t+X(sm)Y(t), which is consistent with that of *N. confucianus* from Shandong as described by Wang et al. (1997, 2003). The karyotype of *N. confucianus* in this study is 2n = 46, FN = 59, 6m+4sm+2st+32t+X(sm)Y(t), indicating that *N. sacer* differs from *N. confucianus* in karyotype. Cytogenetic evidence also supports *N. sacer* as a distinct and valid species.

### Phylogenetic evaluation

The phylogenetic tree results showed that *N. confucianus* species complex is mainly divided into four clades. The first clade is *N. confucianus*, which is found in central China, extending from the northeast to southwest of China; The second clade is *N. sacer*, which is endemic to Shandong; The third clade is *N. lotipes*, which is distributed in the southeast of China; The four clade is distributed in southwestern Yunnan and southeastern Tibet, which has been considered as a new combination, *N. pianmaensis*Ge et al. 2018b. The range of the four species are consistent with Ge et al. (2018a, b). Ge et al. (2018a, b) conducted molecular phylogeny analysis of a species complex of common wild rat species in China and observed the historical dynamics of *N. confucianus* based on coalescence models. It was predicted that *N. c. sacer* was not a subspecies of *N. confucianus*, but rather *N. bukit*, and no *N. confucianus* was found in Shandong. The present study demonstrates that *N. sacer* is a valid species distributed only in Shandong.

The minimum genetic distance (K2P) between the four clades in this study was 0.053. The results of ABGD species delimitation showed that *N. confucianus* are divided into seven groups which are consistent with the phylogenetic tree.

In this study, *N. sacer* was found to be a relatively recent divergence from *N. confucianus*, which differs from the results of Zhang et al. (2016) and Ge et al. (2018a, b). We used more specimens from the east hills branch and southwest mountains branch of *N. sacer*, which also indicated that different datasets had significant effects on the systematic evolution relationship analysis within the genus *Niviventer*.

In addition, the phylogenetic findings in this study are similar to those of Lu et al. (2015) and Zhang et al. (2016), who observed paraphyly among *N. andersoni* and *N. excelsior*, and *N. fulvescens* and *N. huang*. *Niviventer fulvescens* and *N. huang* form a clade in the phylogenetic tree; genetic distance within the clade is 0.019 and these species formed a group according to the ABGD method. Therefore, *N. huang* may require synonymization with *N. fulvescens*. The relationships and taxonomic status of these species require further investigation.

## Conclusions

According to molecular phylogenetic tree and genetic distance, chromosome, and morphology analyses, we found that *N. sacer* should be considered as a distinct species rather than as a subspecies of *N. confucianus* or *N. bukit*. Is speciation from *N. confucianus* should be further examined. *Niviventer sacer* is distributed in the mountains and hills throughout Shandong. *Niviventer confucianus* is also distributed in Shandong, but its distribution is limited to the Southwest mountain areas, which are sympatry of *N. sacer* and *N. confucianus*. The genetic distance (K2P) between these groups is more than 5%, and karyotype and morphology analysis showed significant differences. Thus, it is likely that no hybridization occurs between these species. This study clarifies the taxonomic status of species, thereby enriching biodiversity and improving the species determination of small mammals in China.

## Supplementary materials

Supplementary material 1 Tables S1. Sampling and Genbank sequences information Data type species data Explanation note **Table S1–1.** Sample information of *Niviventer confucianus* collected in the present study, **Table S1–2.** Sample information of genus *Niviventer* used in the present study. https://binary.pensoft.net/file/441958This dataset is made available under the Open Database License (http://opendatacommons.org/licenses/odbl/1.0/). The Open Database License (ODbL) is a license agreement intended to allow users to freely share, modify, and use this Dataset while maintaining this same freedom for others, provided that the original source and author(s) are credited.Yaoyao Li, Yiqiao Li, Haotian Li, Jing Wang, Xiaoxiao Rong, Yuchun Li

Supplementary material 2 Figure S1–S7, Tables S2–S6. Partial morphological and molecular results Data type morphological and molecular data Explanation note **Figure S1.** Landmark (●, red) and semi-landmark (▲, blue) locations in this study (M11107); the scale is 0.5 cm. **Figure S2.** Phylogenetic analyses of *Cytb* gene from all haplotypes by Bayesian Inference. **Figure S3.** Phylogenetic analyses of *Cytb* gene from all haplotypes by neighbor-joining. **Figure S4.** Results of automatic barcode gap discovery (ABGD) analyses. a: Histogram of genetic distance and frequency; b: Line diagram of genetic distance and total number. **Figure S5.** Cluster analysis based on external and skull morphological indices of *N. confucianus*, *N. sacer*, and *N. lotipes*. **Figure S6.** Superimposition of dorsal view (a), ventral view (b), lateral view of skull (c), and lateral view of mandible (d) of *Niviventer confucianus*. **Figure S7.** Comparison of the tail of *N. confucianus* (A), *N. sacer* (B), *N. lotipes* (C). **Table S2.** Results of automatic barcode gap discovery (ABGD) analyses. **Table S3.** One-sample Kolmogorov-Smirnov Normal test of external and craniodental measurements of *N. sacer*, *N. bukit*, *N. confucianus*, and *N. lotipes*. **Table S4.** Analysis of variance (ANOVA) of external and craniodental measurements of *N. sacer*, *N. bukit*, *N. confucianus*, and *N. lotipes*. Values in bold show significant differences among the four species. **Table S5.** Morphological difference between species as determined by LSD tests. Values in bold show significant differences between two taxa. **Table S6.** Discriminant analysis classification of external/skull morphological indices. https://binary.pensoft.net/file/441959This dataset is made available under the Open Database License (http://opendatacommons.org/licenses/odbl/1.0/). The Open Database License (ODbL) is a license agreement intended to allow users to freely share, modify, and use this Dataset while maintaining this same freedom for others, provided that the original source and author(s) are credited.Yaoyao Li, Yiqiao Li, Haotian Li, Jing Wang, Xiaoxiao Rong, Yuchun Li
