# Supplementary material for: Niviventer confucianus sacer (Rodentia, Muridae) is a distinct species based on molecular, karyotyping, and morphological evidence
Source: Zookeys. 2020 Aug 14;959:137–59. doi: 10.3897/zookeys.959.53426 (PMC7442754; doi:10.3897/zookeys.959.53426)
Supplement: Supplementary material 2 — Figure S1–S7, Tables S2–S6. Partial morphological and molecular results [file zookeys-959-137-s002.docx]

**Supplementary Figures and Tables**


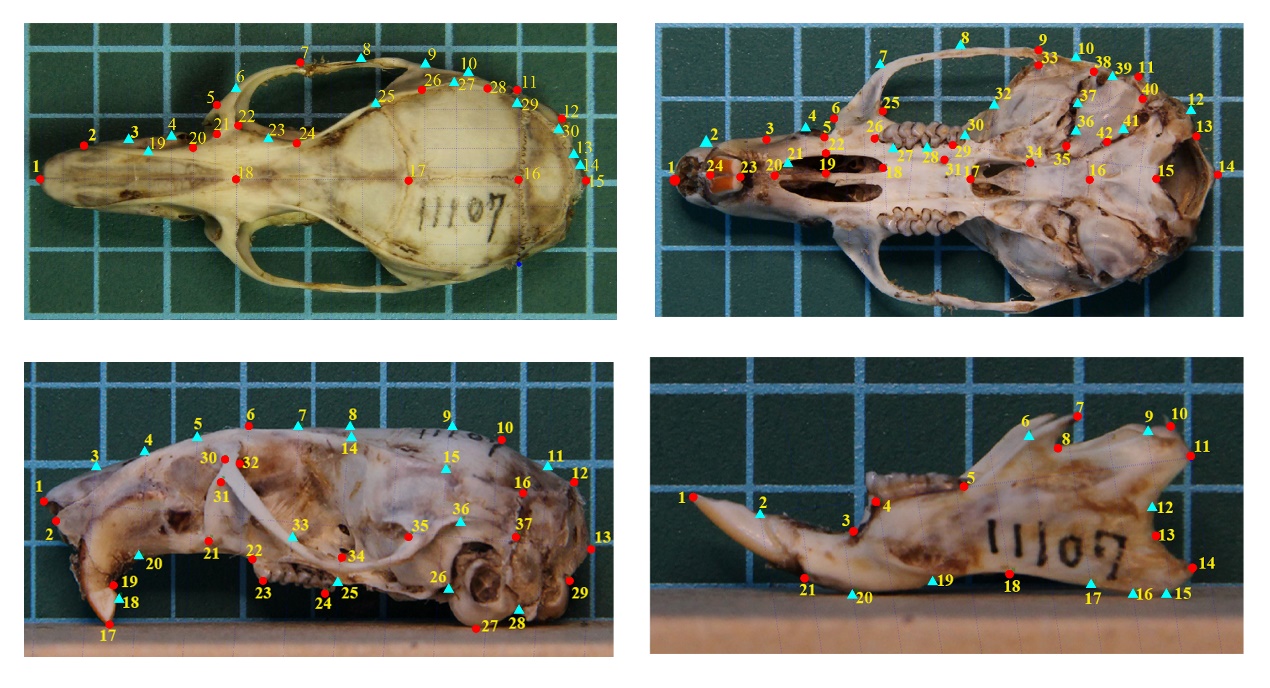


**Supplementary Figure S1** Landmark (, red) and semi-landmark (▲,blue) locations in this study (M11107); the scale is 0.5 cm.


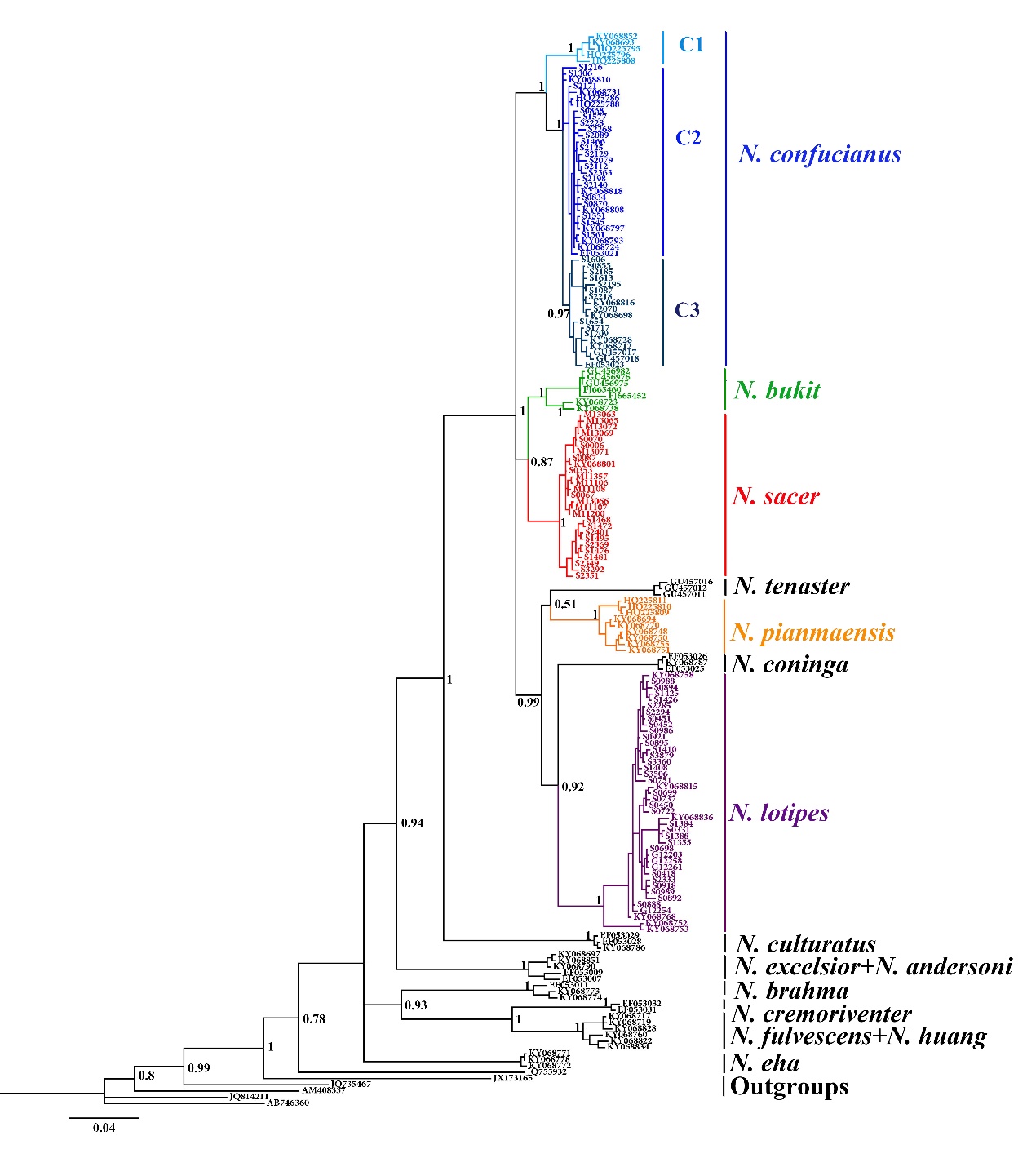


**Supplementary Figure S2** Phylogenetic analyses of *Cytb* gene from all haplotypes by bayesian inference.


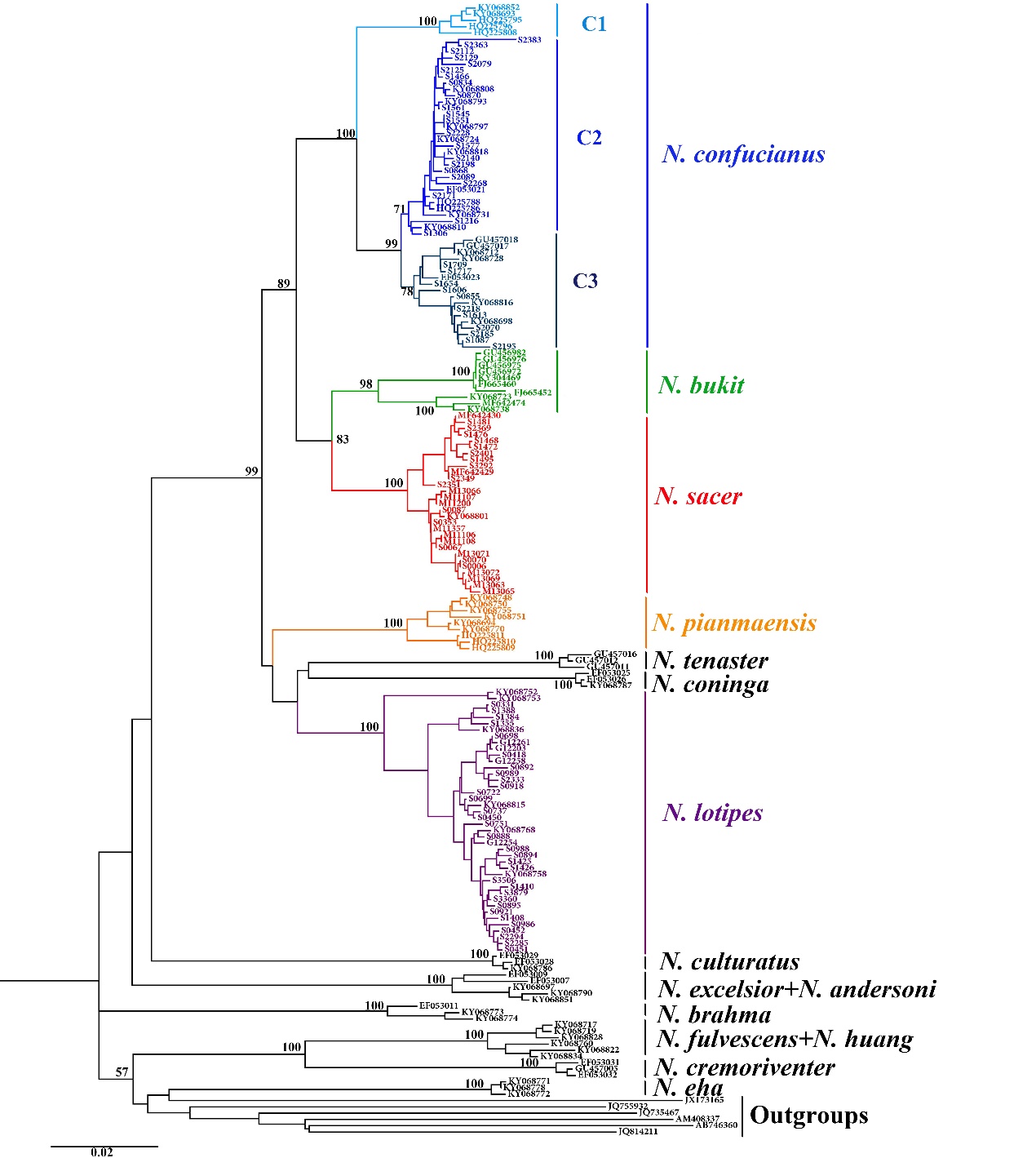


**Supplementary Figure S3** Phylogenetic analyses of *Cytb* gene from all haplotypes by neighbor-joining.


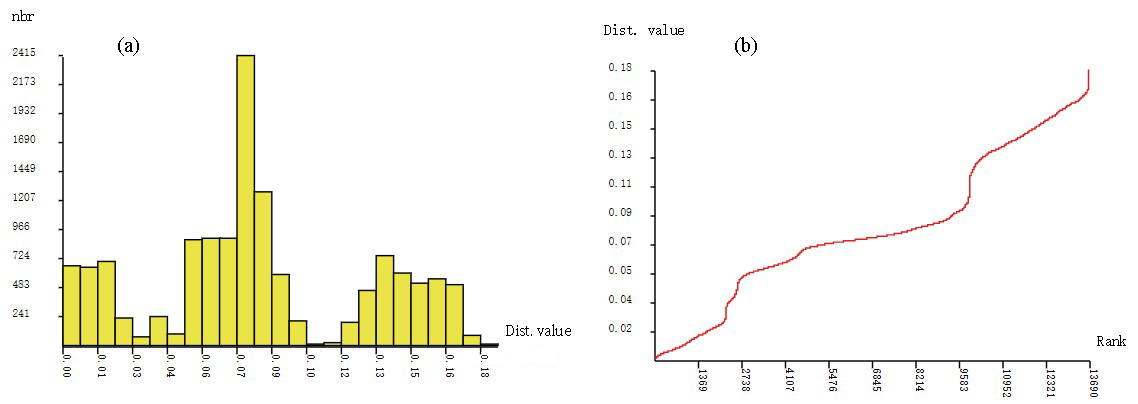


**Supplementary** **Figure S4** Results of automatic barcode gap discovery (ABGD) analyses. a: Histogram of genetic distance and frequency; b: Line diagram of genetic distance and total number.


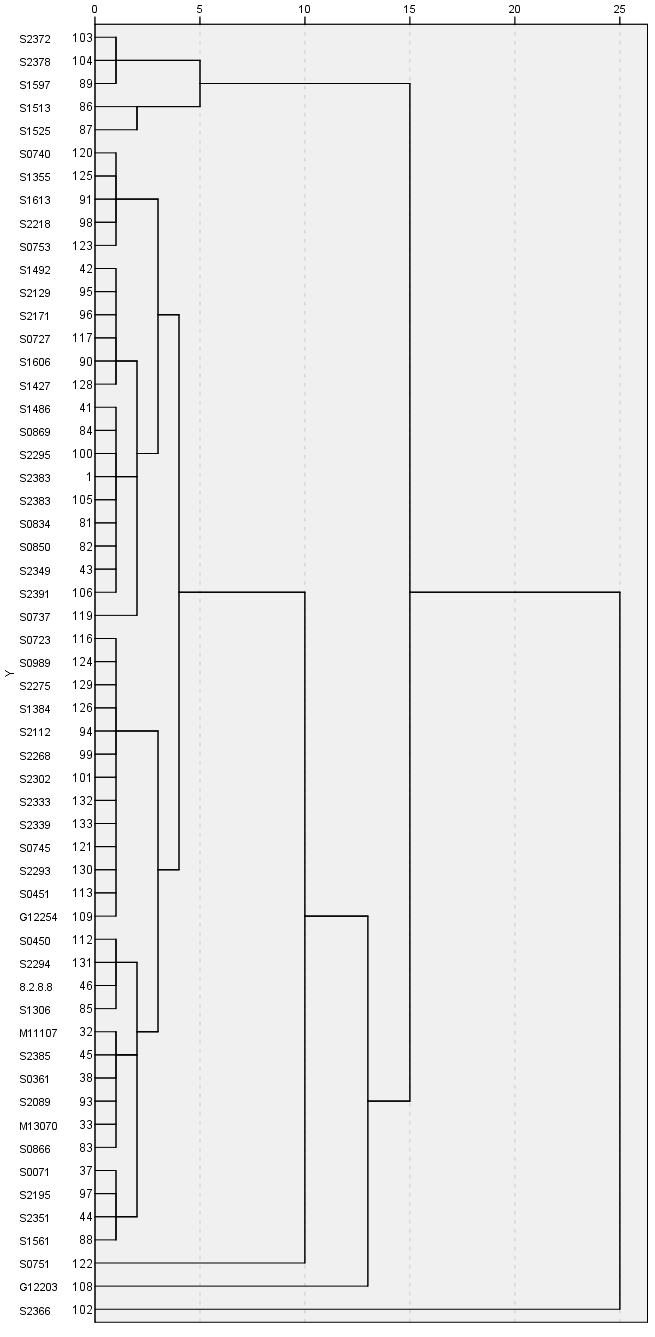


**Supplementary Figure S5** Cluster analysis based on external and skull morphological indices of *N. confucianus*, *N. sacer*, *N. lotipes*.


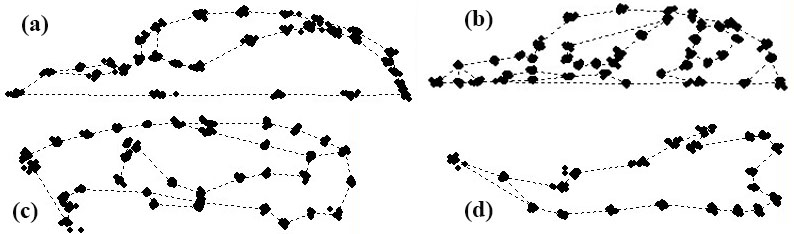


**Supplementary** **Figure S6** Superimposition of dorsal view (a), ventral view (b), lateral view of skull (c), and lateral view of mandible (d) of *Niviventer confucianus*.


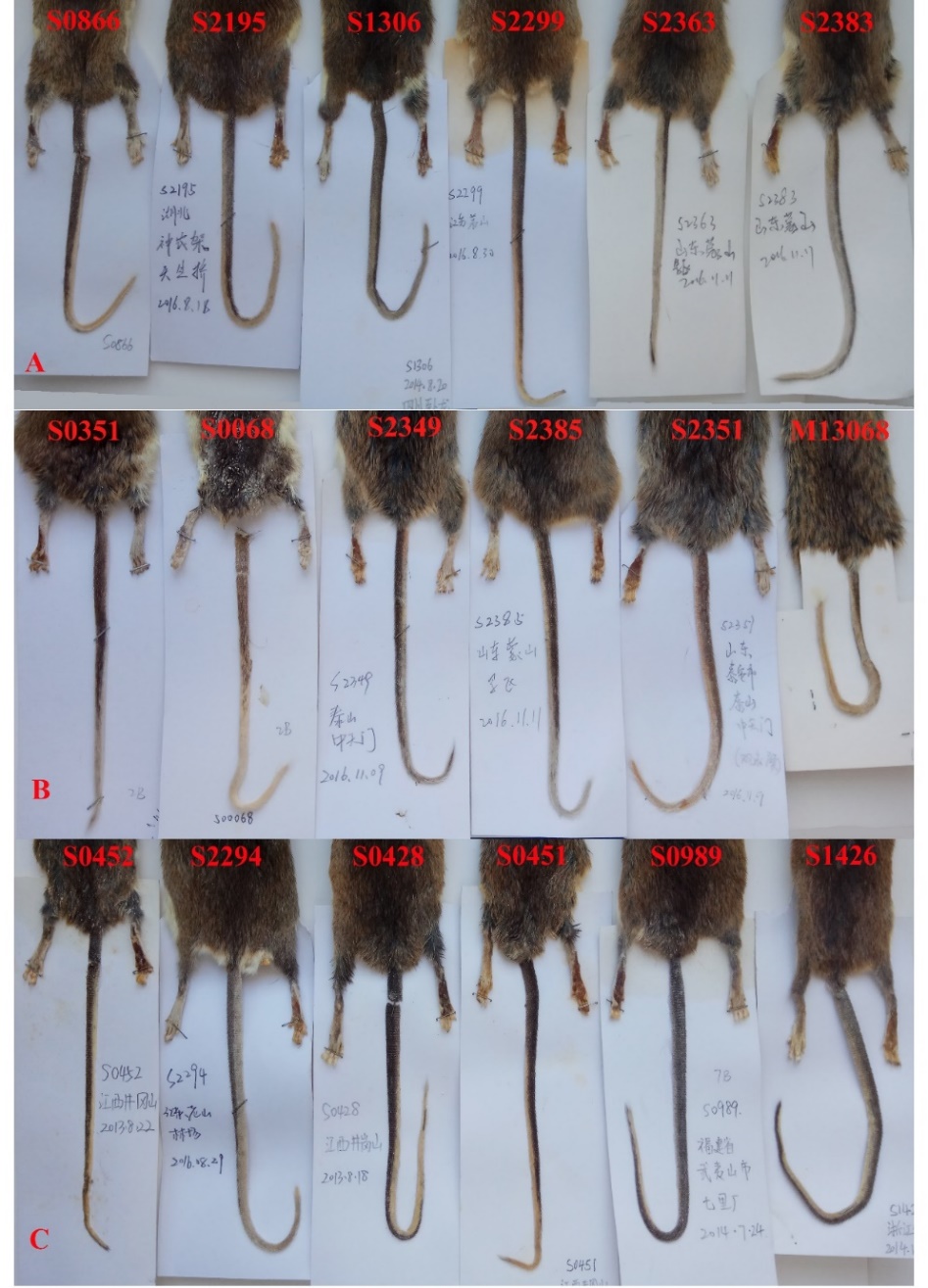


**Supplementary Figure S7** Comparison of the tail of *N. confucianus* (A), *N. sacer* (B), *N. lotipes* (C).

**Supplementary** **Table S2** Results of automatic barcode gap discovery (ABGD) analyses.

| P_min_/P_max_ | X | Partition | Prior intraspecific divergence (P) | | | | | | | | |
| --- | --- | --- | --- | --- | --- | --- | --- | --- | --- | --- | --- |
|  |  |  | 0.001 | 0.0018 | 0.0032 | 0.038 | 0.0056 | 0.083 | 0.01 | 0.0261 | 0.0316 |
| 0.001/0.1 | 0.5 | Initial | 17 | 17 | 17 | 17 | 17 | 17 | 12 | 10 | 6 |
|  |  | Recursive | 43 | 20 | 19 | 19 | 18 | 18 | - | - | - |
|  | 1 | Initial | 17 | 17 | 17 | 17 | 17 | 17 | 12 | 6 | 6 |
|  |  | Recursive | 27 | 20 | 19 | 19 | 18 | 18 | - | - | - |
|  | 1.5 | Initial | 17 | 17 | 17 | 17 | 17 | 17 | 12 | 7 | 0 |
|  |  | Recursive | 19 | 19 | 19 | 19 | 18 | 18 | - | - | 1 |
|  | 2 | Initial | 17 | 17 | 17 | 17 | 17 | 17 | 7 | 7 | 0 |
|  |  | Recursive | 19 | 19 | 19 | 18 | 18 | 18 | 8 | - | 1 |
|  | 2.5 | Initial | 17 | 17 | 17 | 17 | 17 | 17 | 7 | 7 | 0 |
|  |  | Recursive | 18 | 18 | 18 | 18 | 18 | 18 | 8 | - | 1 |

**Supplementary** **Table S3** One-sample Kolmogorov-Smirnov (K-S) test of external and craniodental measurements of *N. sacer*, *N. bukit*, *N. confucianus* and *N. lotipes*.

| Indices | *n* | Mean ± *SD* | *Z* | *P* |
| --- | --- | --- | --- | --- |
| HBL | 121 | 142.84±14.74 | 0.053 | 0.200 |
| TL | 105 | 164.60±17.25 | 0.074 | 0.196 |
| EL | 123 | 20.63±1.52 | 0.075 | 0.086 |
| HFL | 124 | 27.49±1.62 | 0.078 | 0.064 |
| LS | 72 | 36.19±1.72 | 0.073 | 0.200 |
| ZW | 98 | 16.53±0.90 | 0.052 | 0.200 |
| IOB | 98 | 5.51±0.26 | 0.091 | 0.045 |
| BR | 98 | 6.18±0.42 | 0.054 | 0.200 |
| LIF | 98 | 6.48±0.63 | 0.055 | 0.200 |
| LUTR | 72 | 5.88±0.29 | 0.096 | 0.095 |
| LAB | 97 | 5.28±0.39 | 0.117 | 0.002 |
| LD | 98 | 9.37±0.64 | 0.048 | 0.200 |

**Supplementary** **Table S4** Analysis of variance (ANOVA) of external and craniodental measurements of *N. sacer*, *N. bukit*, *N. confucianus* and *N. lotipes*. Values in bold show significant differences among the four species.

|  | Mean Square | *F* | *P* |
| --- | --- | --- | --- |
| HBL | 582.903 | 2.803 | **0.043** |
| TL | 2856.145 | 12.886 | **<0.001** |
| EL | 14.014 | 6.986 | **<0.001** |
| HFL | 17.577 | 7.841 | **<0.001** |
| LS | 1.611 | 0.532 | 0.662 |
| ZW | 3.035 | 4.099 | **0.009** |
| IOB | 0.798 | 16.991 | **<0.001** |
| BR | 0.350 | 2.059 | 0.111 |
| LIF | 6.118 | 28.057 | **<0.001** |
| LUTR | 0.551 | 8.975 | **<0.001** |
| LAB | 0.412 | 2.917 | **0.038** |
| LD | 1.825 | 4.952 | **0.003** |

**Supplementary** **Table S5** Morphological difference between species as determined by LSD tests. Values in bold show significant differences between two taxa.

| species | measurements | *N. sacer* | *N. buki*t | *N.* *confucianus* | *N.* *lotipes* |
| --- | --- | --- | --- | --- | --- |
| *N. sacer* | HBL | — | **0.020** | 0.377 | 0.411 |
|  | TL | — | 0.103 | 0.846 | **<0.001** |
|  | HFL | — | **<0.001** | 0.664 | 0.862 |
|  | EL | — | 0.364 | **<0.001** | **0.005** |
|  | LS | — | 0.666 | 0.246 | 0.689 |
|  | ZW | — | 0.183 | 0.675 | 0.536 |
|  | IOB | — | 0.619 | **<0.001** | **0.013** |
|  | BR | — | 0.848 | 0.397 | **0.016** |
|  | LIF | — | **<0.001** | **<0.001** | **<0.001** |
|  | LUTR | — | **0.033** | 0.137 | **<0.001** |
|  | LAB | — | **0.001** | **0.004** | **0.008** |
|  | LD | — | 0.955 | **0.020** | 0.476 |
| *N. bukit* | HBL | — | — | 0.163 | 0.176 |
|  | TL | — | — | 0.095 | **0.001** |
|  | HFL | — | — | **0.001** | **<0.001** |
|  | EL | — | — | **<0.001** | **0.001** |
|  | LS | — | — | 0.805 | 0.834 |
|  | ZW | — | — | 0.100 | 0.079 |
|  | IOB | — | — | **0.005** | **0.043** |
|  | BR | — | — | 0.757 | 0.197 |
|  | LIF | — | — | **0.038** | **0.022** |
|  | LUTR | — | — | 0.171 | 0.547 |
|  | LAB | — | — | 0.094 | 0.074 |
|  | LD | — | — | 0.175 | 0.709 |
| *N.* *confucianus* | HBL | — | — | — | 0.989 |
|  | TL | — | — | — | **<0.001** |
|  | HFL | — | — | — | 0.591 |
|  | EL | — | — | — | 0.468 |
|  | LS | — | — | — | 0.379 |
|  | ZW | — | — | — | 0.808 |
|  | IOB | — | — | — | 0.101 |
|  | BR | — | — | — | 0.058 |
|  | LIF | — | — | — | 0.657 |
|  | LUTR | — | — | — | **<0.001** |
|  | LAB | — | — | — | 0.810 |
|  | LD | — | — | — | 0.059 |

**Supplementary Table S6** Discriminant analysis classification of external/skull morphological indices.

|  |  | group | Prediction group member | | |  | total |
| --- | --- | --- | --- | --- | --- | --- | --- |
|  |  |  | *N. sacer* | *N. bukit* | *N. confucianus* | *N. lotipes* |  |
| Initial grouping | count | *N. sacer* | 23/13 | 6/0 | 5/2 | 4/0 | 38 |
|  |  | *N. bukit* | 2/0 | 15/3 | 1/0 | 2/1 | 20 |
|  |  | *N. confucianus* | 3/2 | 3/1 | 14/19 | 5/5 | 25 |
|  |  | *N. lotipes* | 0/0 | 0/0 | 2/3 | 19/22 | 21 |
|  | % | *N.* *sacer* | 60.5/86.7 | 15.8/0.0 | 13.2/13.3 | 10.5/0.0 | 100 |
|  |  | *N. bukit* | 10.0/0.0 | 75.0/75.0 | 5.0/0.0 | 10.0/25.0 | 100 |
|  |  | *N. confucianus* | 12.0/7.4 | 12.0/3.7 | 56.0/70.4 | 20.0/18.5 | 100 |
|  |  | *N. lotipes* | 0.0/0.0 | 0.0/0.0 | 9.5/12.0 | 90.5/88.0 | 100 |
